# Provision of Hospital Accommodation

**Published:** 1921-02-19

**Authors:** 


### PROVISION OF HOSPITAL ACCOMMODATION.

By AN INFIRMARY MEDICAL SUPERINTENDENT.

### III. A Solution.

A scheme for the improvement of the medical service of the Poor-law infirmaries which has many advocates is some form of affiliation of them to the voluntary hospitals so that the medical staff of the hospital and its students, if it is a medical school, may help in the investigation and treatment of the patients of the infirmary. Such a system is already working at the Paddington Infirmary. In another London parish a similar system was proposed, but broke down owing to irreconcilable differences between the medical staffs of the two institutions. The system has many advantages—to the patients from the fuller investigation and treatment of their complaints rendered possible by the more varied equipment of the hospital in material and staff, to the students and junior members of the staff from the investigation of diseases seldom seen in the hospital wards, to the senior members of the staff from the knowledge they would gain of the end results of some of their treatment.

### Tendencies and Desirabilities.

But the scheme is not generally applicable. It will work best when an infirmary is linked up to a medical school; and the number of medical schools is small. Many infirmaries are situated at a distance from hospitals, and there it would be difficult to maintain efficient co-operation. The functions of the Poor-law infirmary and of the voluntary hospital are too important for either to be an appanage of the other. Each must develop along its own lines. As things are trending, it seems likely that the infirmary will become the State hospital for those for whom voluntary organisations will not, or cannot, provide accommodation; whilst the hospital will be for those who can afford to pay the whole or the greater part of the cost of their maintenance, or who belong to societies that, in virtue of their contributions, can demand the admission of their members. It is possible that eventually the voluntary hospital will pass into the State system; but it is desirable that it should be kept in existence as long as possible, if only for the advantages that would accrue from the competition of the one class of hospital with the other.

The three great difficulties that must be met before the Poor-law infirmary can deal efficiently with the demands that are now being made upon it, and that will increase if it become a State hospital, are:—(1) The lack of co-operation between individual boards of guardians and between these boards and other bodies dealing with the public provision of medical assistance; (2) the lack of a specialist staff; and (3) the mass of incurable and chronic illness which at present fills up nearly one-half of the beds. The parish is too small a unit for the efficient and economical administration of public medical assistance. We need much larger areas. The ideal system would be to carve the country up into medical districts, the boundaries of which would depend upon the position of the towns within them and the communications and the relations existing between the towns.

Such a redistribution of areas is probably outside the bounds of practical politics, and we must fall back upon a less ambitious scheme—the adoption of the present areas of the counties and county boroughs with some local modifications. In each one there should be an elected body dealing with all the institutions, provided out of the rates of the area for the treatment of bodily and mental disease.

### Classification and Specialisation.

From what has been said about the relation between the workhouse and the infirmary, and from the fact that the workhouses to-day are largely filled with infirm and crippled patients, it follows that this body should also take over most of the workhouses, leaving a few only to deal with the really healthy and able-bodied inmates. It would then be possible to deal efficiently and economically with the patients in the whole area. It is wasteful in money and staff to organise each infirmary on the lines of the voluntary hospital. The number of special cases in each one of them does not justify the expenditure necessary. Within the large areas suggested above the new authority would be able to classify the institutions; to set apart some of them to deal with special classes of disease. Thus one institution might be made a hospital for diseases of the eye, another for diseases of the ear, throat, and nose, a third for nervous diseases, a fourth for rheumatoid arthritis and allied conditions, and so on. These special hospitals would be fully equipped with a specialist staff, and with the apparatus required for thorough investigation and treatment of disease.

Under such conditions, many patients who are now inadequately treated could be saved from drifting into a state of permanent incapacity or, at least, the wage-earning period of their lives could be greatly extended. Take the case of patients with rheumatoid arthritis as an example. At present every infirmary has a number of these patients within its walls. They are most carefully nursed, kept free from bed-sores, and their symptoms are alleviated. But no infirmary is fully provided with the baths and the apparatus for graduated exercises which would retard the progress of the disease; no infirmary is able to carry out the research work which is necessary if the disease is to be controlled and cured. Only a few voluntary hospitals are equipped to deal properly with these patients, and a patient without means, suffering from this disease, drifts early into a condition in

Previous articles appeared on Jan. 29 and Feb. 12, p. 449.

**Provision of Hospital Accommodation**—(*continued*).

which he is a permanent charge upon the community. The State provision of special hospitals for these patients, and, indeed, for all orthopædic conditions, is one of the most urgent necessities of the moment.

How the Scheme would Work.

The scheme here suggested, in brief outline, is as follows. All the infirmaries in one area should be available for any of the sick within that area. This would do away at once with the existing state of things where one infirmary may be overcrowded and that of a neighbouring parish have many empty beds. The infirmary would act as a clearing hospital to which all the sick would be referred in the first instance. Here they would be sorted out and sent to the hospital specialised for their treatment. Patients with ordinary medical and surgical diseases would be treated in the infirmary, incurable patients would be transferred to workhouses equipped and staffed sufficiently to make the rest of their lives as happy as possible, patients with diseases of the eye to eye hospital, orthopædic cases to their special hospitals, phthisical patients to colonies arranged to take the curable, the chronic and the advanced cases, and so on. As time went on an elaborate system capable of dealing with the whole mass of sickness under the best conditions would be evolved. Such a system of specialised hospitals would lead to a higher rate of recovery, and to a reduction in the time under treatment of each patient. Convalescent homes, at present practically non-existent for Poor-law patients, would be established. Many country workhouses, now almost empty, or actually closed, could be adapted for this purpose. This would at once free many beds in the infirmaries for patients acutely ill. And all these advantages could be obtained with very little new building; all that is needed is the classification and adaptation of existing buildings and the organisation for sorting out the patients.

What It Would Involve.

The scheme would require considerable additions to the present medical and nursing staffs. Each infirmary would, as now, be under the control of a medical superintendent. He would give his whole time to the administration of the infirmary and attached buildings. He would control the medical staff, be responsible for statistics and records, arrange the transfer of patients, watch the expenditure and keep down waste, check one ward against another in its use of dressings, food, drugs, and necessaries, and in the average stay of patients within it—in fact be responsible generally for the economical and efficient working of the institution. Under him would be a physician in charge of the medical wards and a surgeon in charge of the surgical wards. Under them again would be the necessary staff of resident medical officers. Specialists would be called in, in consultation, from the special hospitals and from a staff of whole-time specialists available for all the institutions within the area. Each infirmary should have an out-patient department, which would relieve it from the treatment of patients with trivial complaints and of convalescent patients. This department might and should be staffed by a part-time service of general practitioners. In all towns with medical schools, one or more of the public institutions should be linked up with the school, to increase the value of its clinical teaching and to gain for the institutions the value of the work of its students.

I have said nothing about the constitution of the authority that would run this scheme of medical relief. It must be an *ad hoc* authority, a Poor-law body for the whole area, or a Committee of the County Council. Personally I should prefer an *ad hoc* authority. I can conceive a state of society in which the care of the sick would excite as much public interest as a great war, or even the result of a horse-race or a test match. I am afraid, however, that, as things are, an *ad hoc* authority must be ruled out as not practical. A Poor-law authority would suffer from past associations. The voluntary hospitals, owing to their obligations to organised bodies that contribute to their funds and by their demands for payment from patients, are gradually excluding from their wards a large number of the poor who were accustomed to go to them. Many of these people hate the Poor Law and resent the suggestion that they should enter a Poor-law infirmary, and yet it is for these people that fresh hospital accommodation must be found. I think we are driven to the conclusion that the new authority must be a Committee of the County Council.

The scheme thus briefly outlined is not a cheap one. Disease cannot be dealt with on cheap lines. The attempt to do so has crippled the efficiency of our system of Poor-law medical relief. The expenditure would be amply repaid by the increase that would spring from it in national happiness and efficiency.

